# Single ultrasound-guided thoracic paravertebral block with a large volume of anesthetic for microwave ablation of lung tumors

**DOI:** 10.3389/fonc.2022.955778

**Published:** 2022-10-31

**Authors:** Yong Ni, Yulong Zhong, Yue Zhang, Yifei Tao, Jiang Pan, Yiming Zhao, Zhicheng Zhang, Yong Jin

**Affiliations:** ^1^ Pain Department, The Second Affiliated Hospital of Soochow University, Suzhou, China; ^2^ Anesthesia Department, Sichuan Science City Hospital, Mianyang, China; ^3^ The Interventional Therapy Department, The Second Affiliated Hospital of Soochow University, Suzhou, China

**Keywords:** ultrasound, thoracic paravertebral block, microwave ablation, nerve block, lung cancer

## Abstract

**Objective:**

To compare single ultrasound-guided thoracic paravertebral block (TPVB) using a large volume of anesthetic with local anesthesia (LA) in computed tomography (CT)-guided pulmonary microwave ablation.

**Subjects and methods:**

Eighty patients who underwent CT-guided microwave ablation of pulmonary tumors were randomly divided into the TPVB group and the LA group. Patients of the TPVB group were anesthetized with a single injection of a large volume (40 ml) of 0.375% ropivacaine injection at T4, and those of the LA group had local infiltration by the surgeon at the puncture site, and emergency rescue with propofol injection was administered when the patient could not tolerate pain in either group. The following variables were recorded in both groups: general conditions; volume of propofol injection for emergency rescue during ablation; visual analog scale (VAS) scores during ablation and at 0, 2, 12, and 24 h after ablation; the need to use analgesics for rescue within 24 h after ablation; number of ablations; number of punctures performed by the surgeon; patient’s movements during puncturing; and puncturing-associated complications.

**Results:**

Compared with the TPVB group, the amount of emergency use of propofol injection was significantly more in the LA group (*P* < 0.05). There were no significant differences in the VAS scores recorded intraoperatively and at 0, 2, 12, and 24 h after ablation between the two groups (*P* > 0.05). There was a significant difference in the patient’s movements upon puncturing between the two groups (*P* < 0.05), but there were no significant differences in the numbers of punctures and ablations between the two groups (*P* > 0.05). The number of patients using analgesics within 24 h after the operation was also more in the LA group than in the TPVB group, and the difference between the two groups was statistically significant (*P* < 0.05).

**Conclusion:**

Single ultrasound-guided TPVB with a large volume of anesthetic offers effective analgesia for microwave ablation of lung tumors, helping the patient cooperate with the operating surgeon to reduce injury from multiple lung punctures. Further studies are recommended to validate these findings.

## 1. Introduction

**Figure 1:**
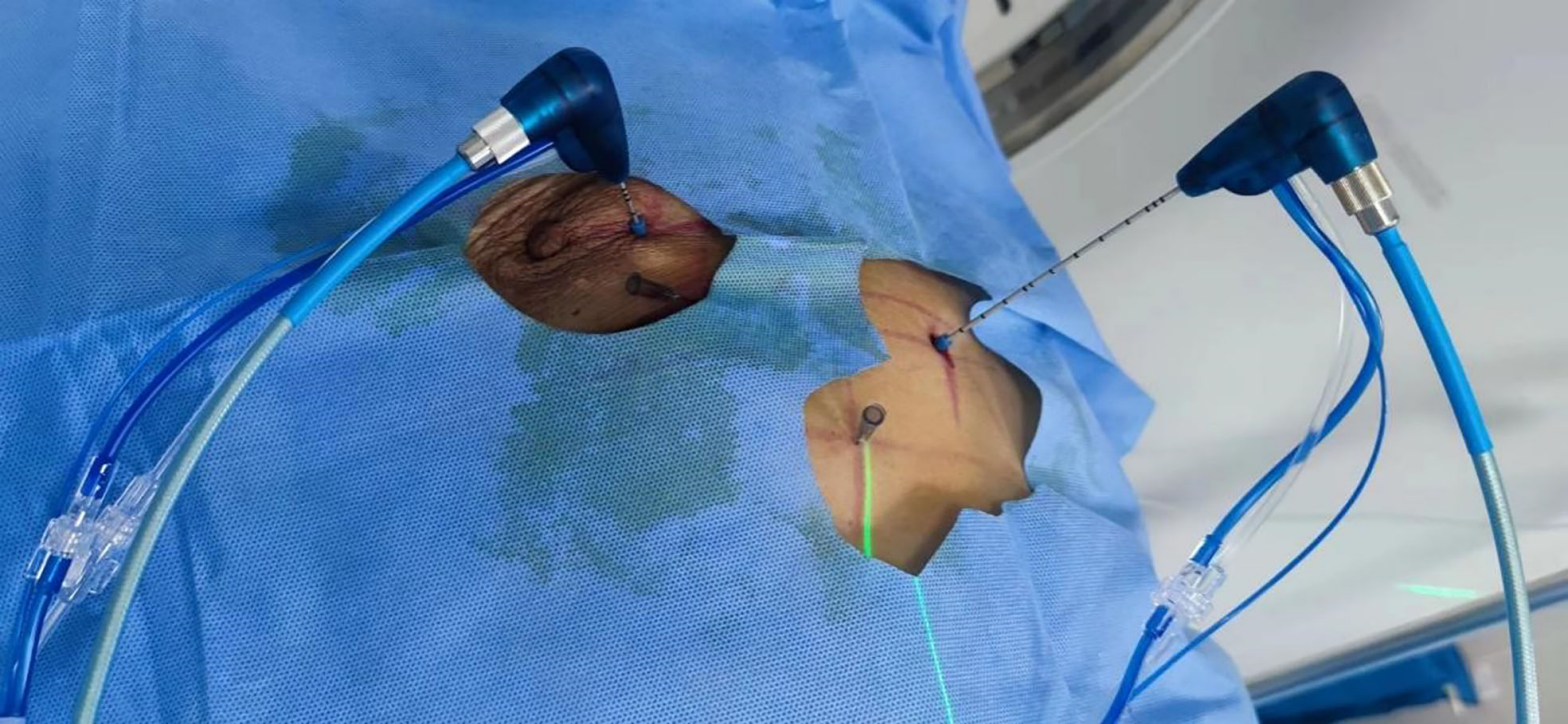
CT-guided microwave ablations of lung tumor.

Microwave ablation (MWA) is the application of electromagnetic waves with frequencies ≥9.2 × 10^8^ Hz to treat solid tumors. By causing oscillation of polar water molecules, microwaves produce frictional heating and ultimately induce cellular death *via* coagulation necrosis. In recent years, MWA has become the preferred thermal ablation method (1), and MWA of lung tumors has shown good efficacy in lung metastases from colorectal cancer (2, 3) and thyroid cancer (4), in percutaneous pulmonary ablation for multiple pulmonary nodules (5), and in non-small cell lung cancer (6, 7) (**Figure 1**). However, MWA of lung tumors causes pain and discomfort during and after ablation; thus, the selection of an anesthetic modality is important. For MWA of lung tumors, the common anesthetic modalities include general anesthesia, local infiltration anesthesia, and nerve block. General anesthesia is an effective analgesic but also has some disadvantages. For instance, under general anesthesia, the patient cannot cooperate with the surgeon, which causes inconvenience to the surgeon.

Since Hara et al. reported ultrasound-guided thoracic paravertebral nerve block (TPVB) in 2007 (8), it has been widely used in thoracic surgery such as in thoracoscopic lung surgery (9), breast surgery (10), rib surgery, and mediastinal surgery and thoracotomies. TPVB has a good analgesic effect in these surgeries. In 2018, Ruscio et al. (11) reported the use of TPVB in computed tomography (CT)-guided percutaneous radiofrequency ablation of lung metastases, with the anesthesiologist performing CT-guided TPVB using 20 ml of 0.375% ropivacaine injection and 2.7 μg/ml of iodixanol. It was concluded that CT-guided TPVB was an effective, low-risk strategy that provided high-quality analgesia. However, CT-guided thoracic paravertebral block is not convenient and only used for analgesia in this study, and patients still need to complete the operation under general anesthesia, which makes patients still unable to cooperate with the surgeon during the operation.

Therefore, we hypothesized that TPVB alone could potentially keep the patient awake to cooperate with the surgeon and also achieve analgesia in MWA of lung tumors. Based on this hypothesis, the current study aimed to compare the intra- and postoperative pain in patients under ultrasound-guided TPVB using a single injection of 40 ml of local anesthetic administered at the T4 level with that of local infiltration anesthesia. This would provide a basis for TPVB alone to be used in MWAs of lung tumors and provide an effective and convenient anesthetic modality for the surgeon and patient.

## 2. Methods

### 2.1 Study design, settings, and participants

In accordance with the Declaration of Helsinki (National Health Council Resolution 196/96), written informed consent was obtained from all patients prior to participation in this randomized, parallel-group, clinical trial, and the trial was approved by the Ethics Committee of the Second Affiliated Hospital of Suzhou University (Ethics Committee JD-LK-2022-042-01). A total of 80 patients who underwent CT-guided MWA of lung tumor [age 29–88 years, body mass index (BMI) 16.61–29.38 kg/m^2^, American Society of Anesthesiologists (ASA) class I–III] were enrolled in the Interventional Department of the Second Affiliated Hospital of Suzhou University.

The inclusion criteria were as follows: 1) patients intended to undergo CT-guided MWA of lung tumor nodules or tumors, 2) age ≥18 years, and 3) ASA class I–III. The exclusion criteria include the following: 1) allergy to anesthetics, 2) long-term opioid use, 3) puncture site infection, 4) difficulty in communication, and 5) allergy to iodinated contrast media. The elimination criteria were 1) subjects who failed to comply with the established study protocol and 2) subjects with incomplete study data.

### 2.2 Sample size justification

The sample size for this study was determined according to the results of a pilot study. This study was a randomized, double-blind, controlled parallel-group trial. The intervention group was the TPVB group, while the control group was the LA group. The primary outcome was the volume of propofol injection used for emergency rescue during ablation. Based on the results of the pilot study, the volume of propofol injection used was 96 ± 35.777 ml in the LA group and 50 ± 70.711 ml in the TPVB group. The following statistics were adopted: *α* = 0.05 (bilateral), power = 0.90, and N1 = N2. The sample size was calculated by using PASS 15 software to be N1 = N2 = 33. Assuming a rate of loss to follow-up of 10%, a sample size of N1′= N2′= 33 ÷ 0.9 = 37 was required. A total of 80 patients were finally enrolled in the two groups.

### 2.3 Randomization

A patient was assigned to the TPVB group or the LA group by using a computerized random-number generator. Opaque envelopes containing a set of materials for either group were prepared prior to subject enrollment, which were sealed and numbered sequentially. After a participant agreed to participate in the trial, the next envelope in the sequence was opened by the coordinator who was not involved in the patient intervention.

### 2.4 Blinding

To control for possible measurement bias and high placebo effect due to active treatment in this study, the following measures were taken: 1) patients discussed their relevant treatment strategies with their treating doctor only, but not with the investigator; 2) two licensed anesthesiologists managed the anesthetic process for the patient; 3) two independent assessors instructed the patient on and administered VAS scoring in the trial; 4) two independent assessors tested the levels of skin hypoesthesia in patients who were intervened; and 5) two investigators who did not participate in the patient assessment were responsible for the blinding and randomization processes.

### 2.5 Interventions

#### 2.5.1 Ultrasound-guided TPVB localization and puncturing

The patient was placed in the prone position. Ribs 1 to 4 were identified from top to bottom by using a low-frequency ultrasound probe (Vinno Technology, Suzhou, China) placed next to the spine, and the fourth rib was marked with a marker pen.

#### 2.5.2 Puncturing

**Figure 2:**
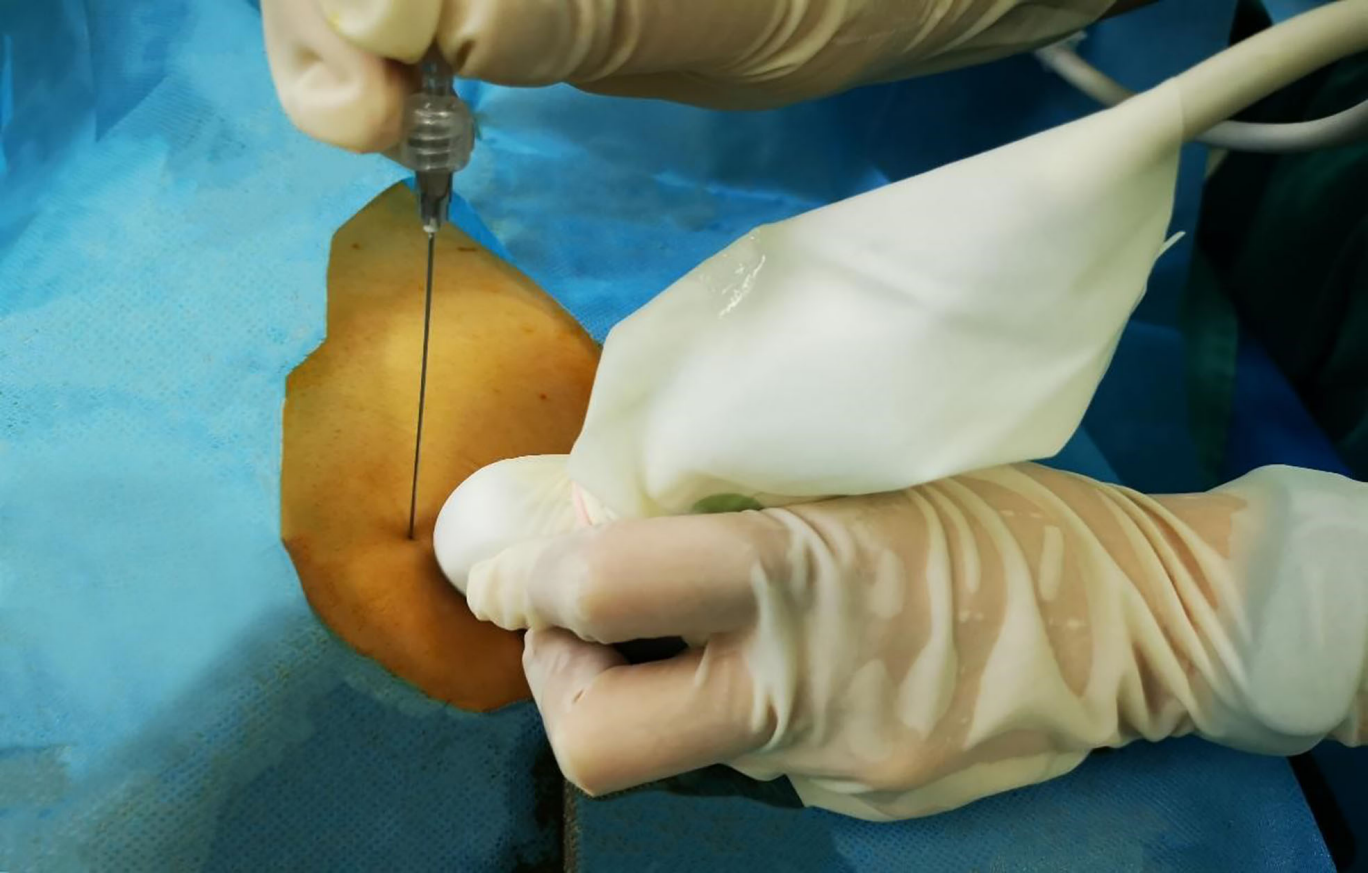
Ultrasound-guided paravertebral puncturing.

**Figure 3:**
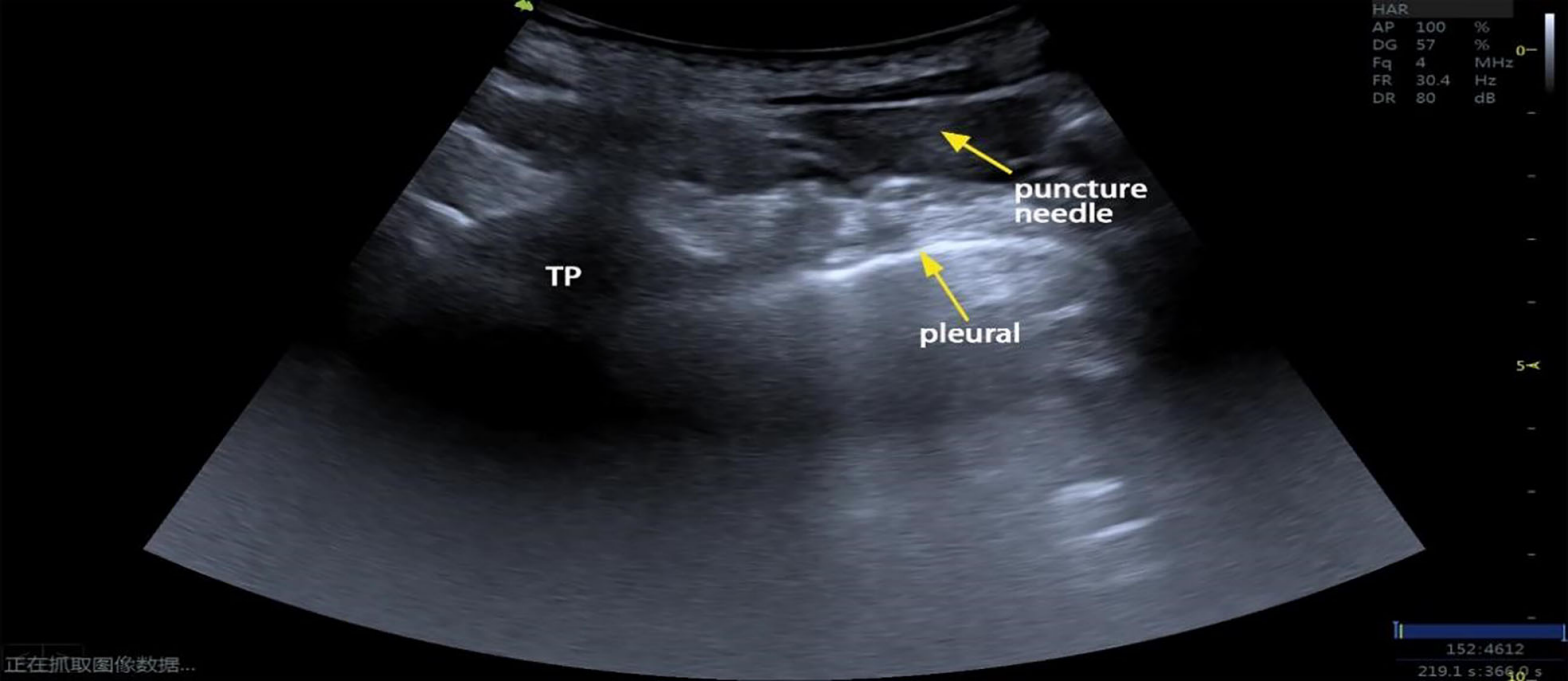
Echocardiography of ultrasound-guided TPVB (TP, transverse process).

At the fourth rib, the low-frequency ultrasound probe was paralleled to the short axis of the body, and the transverse process and pleura were located and identified in this plane. Using the planar technique, the needle was inserted from the lateral side to the medial side and advanced until it entered the triangle formed by the parietal pleura (anterior), the intercostal membrane, and the intercostal innermost muscle (posterior) **Figure 2**. The needle tip and puncture path were clearly displayed during the puncturing process. The puncture needle was advanced to the vicinity of the pleura. The water separation technique was used to observe the pressure beneath the pleura, and the syringe plunger was withdrawn if pressure was present beneath the pleura. If no air or blood was drawn, 40 ml of 0.375% ropivacaine was injected. If there was no pressure beneath the pleura, the puncture needle was adjusted until pressure beneath the pleura was observed; the remaining steps were then performed **Figure 3**.

To ensure the consistency of the puncturing procedure throughout the trial, ultrasound-guided TPVB was performed by one anesthesiologist experienced in nerve block.

### 2.6 Supplementary analgesic use

Rescue with propofol injection for anesthesia and analgesic use for pain relief were allowed after ablation in all patients. Analgesics were used in the following order: non-steroidal anti-inflammatory analgesics, weak opioids, and strong opioids. The use of any analgesics within 24 h after ablation was recorded.

### 2.7 Instruments and assessments

The visual analog scale (VAS) employed in this study has been widely used clinically. Two independent assessors trained the patient on VAS scoring. Two other independent assessors were trained in testing the plane of skin hypoesthesia after ultrasound-guided TPVB.

### 2.8 Outcomes

#### 2.8.1 Primary outcomes

The volume of propofol injection is required for emergency intraoperative intravenous rescue. Propofol injection at a dose of 1–2 mg/kg was given intraoperatively when the VAS score was ≥4 points to keep the patient unconscious until the end of ablation.

#### 2.8.2 Secondary outcomes

Pain intensity was measured by using a 10-cm VAS. The VAS score ranges from no pain (0) to worst pain (10). The VAS scores were recorded intraoperatively and at 0, 2, 12, and 24 h postoperatively. Intraoperative VAS scoring helped determine the intensity of pain and whether emergency intravenous rescue was required. The VAS scores at 0, 2, 12, and 24 h postoperatively revealed the intensity and duration of pain following ablation and helped analyze the intensity and influencing factors of postoperative pain.

#### 2.8.3 The patient’s reactions to intraoperative puncturing through the pleura

The surgeon required the patient to remain immobile during ablation to reduce lung injury from punctures. The disappearance of this reaction may reduce the number of punctures and indirectly reduce lung injury.

#### 2.8.4 Puncturing-associated complications

These included pneumothorax, bleeding, and local anesthetic poisoning.

### 2.9 Statistical analysis

Statistical analyses were performed by using SPSS 25.0. Data of normal distribution are expressed as mean ± standard deviation. Measurement data were compared by *t*-test. Paired *t*-tests were performed for intragroup comparison. Chi-square tests were conducted to compare enumeration data. Rank-sum tests were performed to compare measurement data of non-normal distribution and ranked data. A *P*-value <0.05 was deemed statistically significant.

## 3. Results

### 3.1 General data

Eighty patients with lung tumors were enrolled and randomized into two groups. Demographic data (age and sex), BMI, and ASA classification are presented in **Table 1**.

**Table 1:** Age, sex, BMI, and ASA classification of the patients.

Variable	LA group	TPVB group	*P*-value
Age (years)	57.33 ± 13.96	57.13 ± 14.69	0.732
BMI (kg/m^2^)	22.47 ± 3.45	22.64 ± 3.09	0.310
ASA (I/II/III)	12/24/4	19/16/5	0.193
Sex (F/M)	17/23	22/18	0.263

### 3.2 Primary outcomes

All 40 patients in the LA group received an emergency rescue with propofol injection (1–2 mg/kg) for intolerable intraoperative pain due to ablation, while only 7 patients in the TPVB group received emergency sedation with propofol injection (**Table 2**).

**Table 2:** Comparison of propofol volume, VAS scores, and use of analgesics within 24 h after ablation between the two groups.

Primary outcome variable	LA group	TPVB group	*P*-value
Volume of propofol (ml)	100 (80)	0 (0)	<0.001
Intraoperative VAS score	1 (6)	2 (5)	0.61
VAS score 0 h after ablation	1 (1)	1 (3)	0.264
VAS score 2 h after ablation	1 (1)	1 (2)	0.425
VAS score 12 h after ablation	0 (1)	0 (1)	0.287
VAS score 24 h after ablation	0 (1)	0 (1)	0.333
Use of analgesics within 24 h after ablation (Y/N)	32/8	6/34	<0.001
Patient’s movements during puncturing (N/Y)	0/40	38/2	<0.001

Values outside the parenthesis: median; values in parenthesis: interquartile spacing.

Intraoperative VAS scoring indicated severe pain in 9 patients, moderate pain in 8 patients, and mild or no pain in 23 patients in the LA group and severe pain in 5 patients, moderate pain in 10 patients, and mild or no pain in 25 patients in the TPVB group. VAS scoring immediately after ablation indicated severe pain in 1 patient, moderate pain in 2 patients, and mild or no pain in 37 patients in the LA group and severe pain in 3 patients, moderate pain in 6 patients, and mild or no pain in 31 patients in the TPVB group. VAS scoring at 2 h after ablation indicated severe pain in 1 patient, moderate pain in 1 patient, and mild or no pain in 38 patients in the LA group and severe pain in 0 patients, moderate pain in 2 patients, and mild or no pain in 38 patients in the TPVB group. VAS scoring at 12 h after ablation indicated severe pain in 1 patient, moderate pain in 3 patients, and mild or no pain in 36 patients in the LA group and severe pain in 3 patients, moderate pain in 0 patients, and mild or no pain in 37 patients in the TPVB group. VAS scoring at 24 h after ablation indicated severe pain in 0 patients, moderate pain in 2 patients, and mild or no pain in 38 patients in the LA group and severe pain in 2 patients, moderate pain in 0 patients, and mild or no pain in 38 patients in the TPVB group. There were no significant differences in the VAS scores recorded intraoperatively and at 0, 2, 12, and 24 h after ablation between the two groups (*P* > 0.05) (**Table 2**).

Thirty-two patients in the LA group and 6 patients in the TPVB group received analgesic rescue within 24 h after ablation, and there was a significant difference between the two groups (*P* < 0.05) (**Table 2**).

Upon puncturing through the pleura, movement was recorded in all 40 patients in the LA group but in only 2 patients in the TPVB group, and this difference between the two groups was significant (*P* < 0.05) (**Table 3**).

**Table 3:** Comparison of patient’s movements, number of nodules ablated, and number of punctures between the two groups.

Secondary outcome variable	LA group	TPVB group	*P*-value
Patient movement upon puncturing (N/Y)	0/40	38/2	<0.001
Number of nodules ablated	1 (1)	1 (1)	0.542
Number of punctures	9 (9)	8 (6)	0.321

Values outside the parenthesis: median; values in parenthesis: interquartile spacing.

The number of nodules ablated per patient differed insignificantly between the LA group and the TPVB group [1 (1) *vs*. 1 (1)] (*P* > 0.05) (**Table 3**).

There were no significant differences in the number of punctures between the LA group and the TPVB group [9 (9) *vs*. 8 (6)] (*P* > 0.05) (**Table 3**).

In the LA group, there were 63 puncture sites across C7 to T9, and 1 patient had an ablation site close to the diaphragm. In the TPVB group, there were 57 puncture sites, and 2 patients had an ablation site close to the pleura (**Table 4**).

**Table 4:** Puncture sites and ablation sites close to the pleura or diaphragm.

LA group	Puncture site	Close to the diaphragm?	Close to the pleura?	TPVB group	Puncture site	Close to the diaphragm?	Close to the pleura?
1	T5, T5, T2, T4	No	No	1	T10	No	No
2	T3	No	No	2	T6	No	No
3	T1	No	No	3	T8, T8	No	No
4	T1, T2	No	No	4	T3	No	No
5	T6, T7	No	No	5	T3	No	No
6	T4	No	No	6	T8	No	No
7	C7	No	No	7	T1	No	No
8	T2	No	No	8	T7	No	No
9	T4, T8	No	No	9	T2, T2	No	No
10	T1	No	No	10	T2	No	No
11	T3	No	No	11	T9	No	No
12	T1	No	No	12	T4	No	No
13	T2, T7	No	No	13	T10	No	No
14	T6	No	No	14	T5	No	No
15	T4	No	No	15	T3, T3, T3	No	No
16	T6, T6	No	No	16	T1	No	No
17	T6	No	No	17	T8, T4	No	No
18	T6, T7	No	No	18	T3	No	No
19	T1, T1	No	No	19	T7	No	No
20	T4, T4	No	No	20	T7, T7	No	No
21	T9	Yes	No	21	T7	No	No
22	T2	No	No	22	T4	No	No
23	T8	No	No	23	T4, T4	No	No
24	T2, T3	No	No	24	T3	No	No
25	T5, T5	No	No	25	T2	No	No
26	T6	No	No	26	T3, T4	No	No
27	T8	No	No	27	T2, T2	No	No
28	T5, T7	No	No	28	T9	No	No
29	T3, T6, T8	No	No	29	T2, T2	No	No
30	T3	No	No	30	T1, T4	No	No
31	T3, T7	No	No	31	T1	No	No
32	T1	No	No	32	T5, T3	No	Yes
33	T6, T8, T9	No	No	33	T1, T2	No	No
34	T3	No	No	34	T2, T6	No	Yes
35	T4	No	No	35	T5	No	No
36	T3, T3, T3	No	No	36	T1, T4	No	No
37	T7	No	No	37	T2, T3	No	No
38	T2, T3	No	No	38	T4	No	No
39	T3	No	No	39	T1, T3	No	No
40	T5, T5	No	No	40	T3	No	No

**Figure 4:**
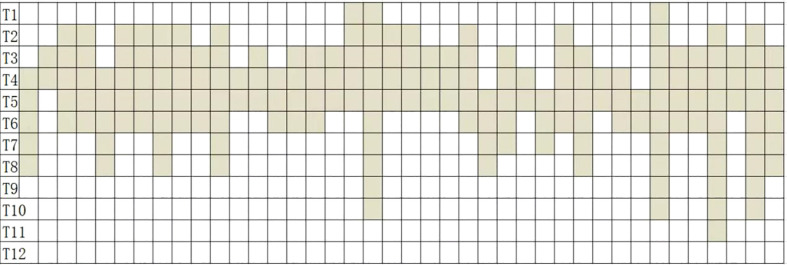
Skin hypoesthesia in 40 patients following ultrasound-guided TPVB.

There was skin hypoesthesia in a minimum of 1 segment and a maximum of 10 segments (an average of 4.625 segments) following ultrasound-guided TPVB (**Figure 4**).

No puncturing-associated complications occurred in 40 patients following ultrasound-guided TPVB.

## 4. Discussion

Pain during MWA of lung tumors predominantly stems from 1) local skin puncture (12); 2) heat transferred to the surrounding tissues during ablation (7); 3) intercostal neuralgia caused by ablation near the pleura, especially near the intercostal nerve (7); and 4) phrenic nerve injury from ablation near the mediastinum or phrenic nerve or referred pain of the shoulder caused by hampered conduction of the phrenic nerve (13). Determining the underlying causes of pain in MWA of lung tumors helps with the selection of appropriate anesthetic modalities and facilitates both intra- and postoperative analgesia. The puncture sites for MWA of lung tumors spanned widely from C7 to T9, and one patient even had more than two MWAs of lung tumor sites; hence, a large volume of ropivacaine (40 ml) was injected at a single site.

There is evidence of some experiences with anesthesia for pulmonary MWA. Kashima et al. used 0.5%–1% lidocaine hydrochloride injection for local infiltration anesthesia in combination with 0.1–0.2 mg of fentanyl for analgesia (14). Hoffman et al. compared general anesthesia with conscious sedation and found that general anesthesia was better for anxious, restless patients and for those unable to hold their breath during needle positioning (15). Ruscio et al. reported the use of ultrasound-guided TPVB in CT-guided pulmonary percutaneous radiofrequency ablations and found that ultrasound-guided TPVB was an effective, low-risk, high-quality modality of analgesia (11). García et al. further demonstrated the advantages of ultrasound-guided TPVB in a case report of pulmonary radiofrequency ablation, suggesting that a single ultrasound-guided TPVB was an effective and safe technique that led to high patient satisfaction and low complication rates. TPVB enables better cooperation between the patient and surgeon while providing good analgesia, which minimizes the risk of pneumothorax by maintaining spontaneous ventilation and has a low failure rate (estimated at 1.98%) (6). In the current study, the volume of intraoperative propofol injection required for emergency rescue was significantly smaller in the TPVB group compared with that in the LA group, which directly indicated that more patients in the TPVB group remained awake during ablation and were better able to cooperate with the surgeon to hold their breath during puncturing (15). Furthermore, compared with the LA group, significantly fewer patients in the TPVB group received emergency analgesic rescue within 24 h after ablation. There were no differences in the VAS scores recorded intraoperatively and at 0, 2, 12, and 24 h after ablation, which were mainly related to the emergency rescue with propofol injection if the intraoperative VAS scores were 4 or more points and to the postoperative analgesic rescue with analgesics. The primary objective of both rescues was to relieve the patient’s pain.

In the present study, no puncturing-associated complications occurred in the 40 patients receiving ultrasound-guided TPVB, which may relate to the skillfulness of the operator and may also relate to the small number of cases. This requires verification by studies with larger sample sizes.

This study does have some limitations. Firstly, a large volume (40 ml) of ropivacaine was injected at a single site on the T4 level. Surgeons select the puncture site according to the location of the patient’s tumor, several sites of the same patient can be punctured at the same time, and different body positions will affect the selection of the ultrasound-guided thoracic paravertebral puncture site. Improvements will be made in future studies to adjust the puncture site for TPVB based on the surgical site and to observe the effect of the surgeon’s puncture site and the puncture site for ultrasound-guided TPVB on the control of ablation-induced pain. Secondly, when lung ablation was in the vicinity of the diaphragm, the pain could not be completely controlled. This is because phrenic nerve block has a certain probability of diaphragm inhibition, and phrenic nerve inhibition will affect the patient’s respiration to some extent. Thirdly, no sedatives were routinely administered in this study. In future studies, consideration will be given to how the use of sedatives can ensure that the patient can cooperate with the surgeon during the procedure.

## 5. Conclusion

Single ultrasound-guided TPVB with a large volume of anesthetic can provide effective analgesia for most MWAs of lung tumors, and patients can better cooperate with the surgeon to reduce injury from multiple lung punctures. Multicenter clinical studies with larger sample sizes are required in the future to validate the findings of the current study.

## Data availability statement

The original contributions presented in the study are included in the article/Supplementary Material. Further inquiries can be directed to the corresponding author.

## Ethics statement

The studies involving human participants were reviewed and approved by Ethics Committee of the Second Affiliated Hospital of Soochow University. The patients/participants provided their written informed consent to participate in this study. Written informed consent was obtained from the individual(s) for the publication of any potentially identifiable images or data included in this article.

## Author contributions

YN and YLZ were responsible for collecting the data and writing the article. YZ was responsible for touching up the article. YT, JP, YMZ and ZZ contributed equally to the writing of the article. YJ was responsible for the overall analysis as well as for reviewing the article. All authors contributed to the article and approved the submitted version.

## Conflict of interest

The authors declare that the research was conducted in the absence of any commercial or financial relationships that could be construed as a potential conflict of interest.

## Publisher’s note

All claims expressed in this article are solely those of the authors and do not necessarily represent those of their affiliated organizations, or those of the publisher, the editors and the reviewers. Any product that may be evaluated in this article, or claim that may be made by its manufacturer, is not guaranteed or endorsed by the publisher.
